# Timut Pepper Extract Slows Age-Dependent Decline of Mobility and Collagen Loss and Promotes Longevity

**DOI:** 10.3390/nu16132122

**Published:** 2024-07-03

**Authors:** Elisabeth Jongsma, Giovanna Grigolon, Julia Baumann, David Weinkove, Collin Y. Ewald, Franziska Wandrey, Torsten Grothe

**Affiliations:** 1 Laboratory of Extracellular Matrix Regeneration, Institute of Translational Medicine, Department of Health Sciences and Technology, ETH Zürich, 8603 Schwerzenbach, Switzerland; 2 Mibelle Group Biochemistry, Mibelle AG, 5033 Buchs, Switzerland; 3 Magnitude Biosciences Ltd., NETPark Plexus, Thomas Wright Way, Sedgefield TS21 3FD, UK; 4 Department of Biosciences, Durham University, Stockton Road, Durham DH1 3LE, UK

**Keywords:** spice, movement, lifespan, cognition, healthspan, supplement, speed, aging, hydroxy-α-sanshool, extracellular matrix

## Abstract

Investigations into human longevity are increasingly focusing on healthspan enhancement, not just lifespan extension. Lifestyle modifications and nutritional choices, including food supplements, can significantly affect aging and general health. Phytochemicals in centenarians’ diets, such as those found in Timut pepper, a Nepalese spice with various medicinal properties, may contribute to their longevity. Similarly, Sichuan pepper, a related species, has demonstrated anti-inflammatory and neuroprotective activities. With the broader purpose of uncovering a novel treatment to address aging and its comorbidities, this study aims to investigate the potential lifespan- and healthspan-promoting effects of Timut pepper using the model organism *Caenorhabditis elegans***.** We show that Timut pepper extract extends *C. elegans’* lifespan at different maintenance temperatures and increases the proportion of active nematodes in their early adulthood. In addition, we show that Timut pepper extract enhances speed and distance moved as the nematodes age. Finally, Timut pepper extract assures extracellular matrix homeostasis by slowing the age-dependent decline of collagen expression.

## 1. Introduction

Human longevity is a success story of the past century, and it by no means has reached its peak. In terms of longevity interventions, the focus in recent years has not only been on extending life expectancy, but importantly also focusing on improving overall health. Whereas lifespan is defined as the number of years lived from birth until death, healthspan describes the time during which a person is healthy within their lifespan [1,2]. This time of wellness is often less than the total years lived, as illness can unfortunately occur early while life continues for many years [3]. Therefore, extending healthspan is a valid strategy to improve overall quality of life and promote longevity. Nutritional choices and lifestyle modifications can have profound impacts on aging and overall health [4]. In this context, food supplements serve as a useful resource for easily incorporating new dietary practices into one’s routine and in multiple cases have been proven to help prevent disease, enhance performance, and support general health [5,6]. In the diets of centenarians (people over the age of 100), phytochemicals contained in vegetables, fruits, and spices may play a significant role [4]. For example, the residents of Okinawa, the southernmost prefecture of Japan, who are known for their long average life expectancy, high numbers of centenarians, and low risk of age-associated diseases, can count many herbs, spices, and flavorings in their cuisine that not only provide enhanced taste to foods but have medicinal properties as well [7]. Timut pepper, *Zanthoxylum armatum*, is a plant member of the citrus family (Rutaceae) that is native to the mountainous regions of Southern Himalaya edges, especially Nepal. Also called prickly ash, this Nepalese pepper has a characteristic grapefruit-like taste and produces a tingling, numbing sensation on the tongue as mediated by interaction with transient receptor potential vanilloid 1(TRPV1) and transient receptor potential cation channel subfamily A member 1 (TRPA1) receptors [8]. Timut pepper is one of the most used spices in Nepalese, Bhutanese, and Tibetan cuisine, as it is traditionally believed to have stomachic properties (promoting appetite or assisting digestion) [9]. Aside from its culinary application, various ethnomedicinal uses of Timut pepper in antipyretic, carminative, antidiabetic, antiasthma, and antirheumatic ailments have been documented [10]. Furthermore, fruit extracts of *Z. armatum* have been shown to exhibit strong antifungal, cytotoxic, phytotoxic, insecticidal, and antileishmanial effects [2]. In addition, several studies have proved the beneficial effects of Sichuan pepper, a closely related species within the genus *Zanthoxylum*, on multiple conditions [11]. Sichuan pepper has been shown to reduce inflammation by modulating the expression levels of toll-like receptor 4 (TLR-4) and cytokine release [12], as well as by regulating NF-kB and PPARγ pathways [13]. Other studies have pointed in the direction of a neuroprotective activity of Sichuan pepper [14,15,16] and, in the context of metabolic health, Sichuan pepper extract has been proved to alleviate hyperlipidemia in murine and atherosclerotic guinea pig models [17], as well as in hamsters fed a high-fat diet [18]. Various phytochemicals, such as lignins, sterols, alkaloids, coumarins, phenolics, terpenoids, and flavonoids, as well as their glycosides and benzenoids, fatty acids, alkenic acids, and amino acids, have been isolated from the Timut pepper plant [19,20,21]. More specifically, linalool, limonene, and methyl cinnamate, as well as the flavonoid tambulin, can be isolated from its seeds [20,21], while the essential oil obtained from dried fruits contains linalool, linalyl acetate, citral, geraniol methyl cinnamate, limonene, and sabinene [20]. Specific biochemical effects have been described for a few of these molecules. For example, linalool and linalyl acetate, as well as the coumarin bergapten, have been described for their anti-inflammatory activity [22,23], whereas the flavonoid 3,5-diacetyltambulin showed significant antibacterial activity against Gram-positive bacteria [24]. In line with this, we aimed to investigate the lifespan- and healthspan-promoting effect of a Timut pepper extract. We did so by testing its ability to extend nematodal lifespan at different maintenance temperatures and by assessing its efficacy in promoting nematodal motility. Furthermore, we tested whether Timut pepper extract could enhance the expression of a collagen-encoding gene, thereby counteracting the age-dependent decline in collagen expression. We used the model organism *C. elegans* for this purpose, given its short life cycle and the high percentage of conserved genes between this nematode and humans [25,26].

## 2. Materials and Methods

### 2.1. Extract Preparation

The dried fruits of Timut pepper were used to prepare the Timut pepper extract by a gentle extraction process, using ethanol, water, and MCT (medium-chain triglycerides) oil as extraction solvent. Gum Arabic was used as a carrier to produce a powder extract. This process ensures a high content of the main lead substance, the alkylamide hydroxy-α-sanshool. The extract, traded as SaraPEPP™ Nu pwd, was provided by Mibelle Group Biochemistry, Switzerland.

### 2.2. Agar Media and Compound Solutions Preparation for Healthspan Assays

For the healthspan assays, a stock solution of Timut pepper powder extract was prepared by dissolving the test compound in water to a concentration of 25 mg/mL. Defined agar media (2% agar) [27] was prepared as described and the Timut pepper extract stock solution was diluted in the agar media (cooled to 55 °C) to a final concentration of 1 mg/mL. The agar-compound-media was then added to 3.5 cm Petri dishes, with 5 mL media per dish. The plates were then allowed to solidify at room temperature.

### 2.3. Assessment of Compatibility with Agar, Escherichia coli Growth, and C. elegans Population Growth

After the plates solidified, they were visually inspected for precipitation and opacity. The plates were then seeded with *E. coli* (strain OP50, Caenorhabditis Genetics Center (CGC), Minnesota) and, after 24 h, bacterial growth and structure were assessed by eye. Four juvenile *C. elegans* (L4 larvae; wild-type strain N2, CGC) were added to each plate at 24 °C. *C. elegans* progeny production and growth were monitored daily for 5 days, which corresponds to 2 generations, while bacterial lawn consumptions were also assessed. If growth and development are unaffected, the lawn is completely consumed after 5 days. As a result, the optimal test concentration of 1 mg/mL Timut pepper extract was defined.

### 2.4. Assessment of Speed and Distance Moved

For the healthspan assessment, 3.5 cm Petri dishes were poured with agar media containing 1 mg/mL Timut pepper extract, water solvent (control), and 16 µg/mL of the positive control sulfonamide-antibiotic sulfamethoxazole (SMX), a compound known to extend health- and lifespan in *C. elegans* [28,29,30]. Twelve Petri dishes were prepared for each condition. After solidification and visual inspection, the healthspan assay was performed according to the following timetable:

- Day −4: Adult temperature-sensitive *C. elegans* of the strain SS104 (SS104 *glp-4(bn2)*) from unstarved cultures were set up to lay eggs overnight at 15 °C on 9 cm Petri dishes.
- Day −2: Gravid *C. elegans* were removed. Then, 3.5 cm dishes with formulations were poured as specified above and left to solidify. *E. coli* OP50 were then added to the center of the Petri dishes (50 µL of culture in LB broth) and allowed to dry (room temperature (RT), 20 °C).
- Day −1: Petri dishes were shifted to 24 °C and *C. elegans* were shifted to 24 °C to induce sterility.
- Day 0: 12 L4 (fourth larval stage) *C. elegans* were picked for each experimental plate. Plates were loaded onto the Wormgazer^TM^. Run was started.
- Day 1: First day of adulthood.
- Day 7: Run was terminated, and Petri dishes were removed and inspected manually for any deviations.

### 2.5. Imaging Data Collection for Movement Assays

The proprietary Wormgazer^TM^ imaging technology records the *C. elegans* that move above a certain threshold distance in a 160 s window. The number of moving *C. elegans* and their speeds (distance travelled per second) is used for analysis. The recording is repeated every 5 min for each Petri dish (12 dishes per condition). The mean number of *C. elegans* moving at any timepoint is analogous to survival metrics in a lifespan assay. The mean speed and speed distributions are an indication of the health and behavior of the *C. elegans* [30].

### 2.6. Lifespan Analysis

A stock solution of 100 mg/mL Timut pepper extract was prepared by dissolving the powder extract in water. The stock solution was then added to 2% Nematode Growth Medium (NGM) (cooled to 55 °C) for a final concentration of 1 mg/mL. The agar-compound-media was then added to 6 cm Petri dishes, with 8 mL media per dish. The plates were then allowed to solidify at room temperature. For this analysis, the *C. elegans* strain TJ1060 *spe-9(hc88)* was used, as these can be sterilized by culturing at 25 °C. The sterilization process assures food availability for the duration of the experiment but does not affect lifespan [31]. Larval stage 1 (L1) animals were plated on 10 cm plates containing the extract, at 25 °C. At day 3 of adulthood, the animals were moved back to 20 °C and 40 *C. elegans* were placed per plate. When the lifespan assay was performed with the lifespan machine (at 20 °C), these plates were kept in “quarantine” at 20 °C to select the non-condensated and non-contaminated plates at day 6 of adulthood to ensure imaging quality. At day 6, four plates were loaded onto the scanners. Every scanner included four water control plates. An image was taken every 2 h for 30 days. Death events were analyzed by the software and verified by visual inspection from the resulting images. For the manual lifespan at 25 °C, four plates were used per compound, and death events were counted once per day.

### 2.7. Collagen Expression Assay

The assay was run as previously published [32]. Briefly, the *C. elegans* strain LSD2002, which contains a green fluorescent protein (GFP) as a marker for expression driven by the collagen *col-144* promoter, was used. Synchronized *C. elegans* at larval stage 1 were cultured on NGM plates or NGM supplemented with different concentrations of Timut pepper extract. On day 4 of adulthood, the expression of *col-144* was scored by imaging and analysis of GFP intensity. The *col-144* expression was read out as GFP intensity per surface area of the animal. Images were taken with an upright brightfield fluorescence microscope (200× magnification). GFP intensity was measured and autofluorescence was subtracted using ImageJ software release 3.1.9. GraphPad Prism 8 was used for data processing.

## 3. Results

### 3.1. Timut Pepper Extract Extends C. elegans’ Lifespan

**Figure 1:**
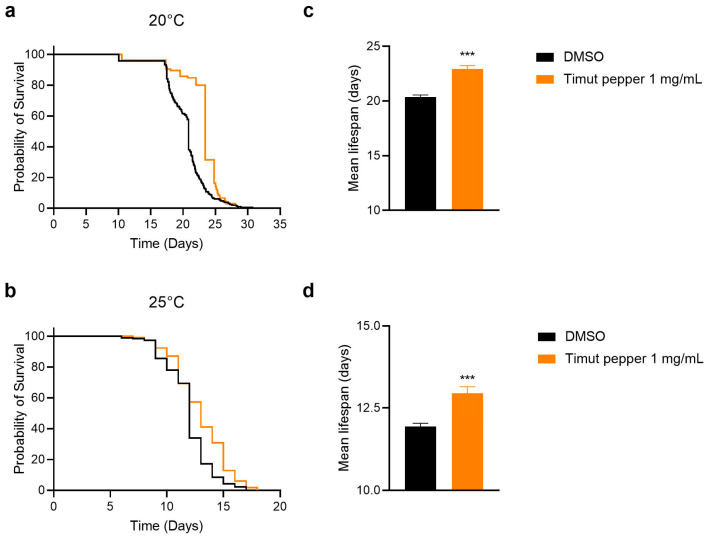
Lifespans of *C. elegans* strain TJ1060 spe-9(hc88) treated with DMSO (vehicle control) or with Timut pepper extract at 20 °C (**a**) and 25 °C (**b**) and mean lifespan thereof (**c**,**d**). Statistical analysis: unpaired *t*-test. *** *p* ≤ 0.001.

Given the numerous health-promoting applications of Timut pepper [10], we sought to investigate whether Timut pepper extract could promote longevity. Supplementing Timut pepper extract (SaraPEPP^TM^ Nu pwd) to *C. elegans* showed a significant mean lifespan-extending effect at a dose of 1 mg/mL at both assay temperatures: 20 °C assayed with the automated lifespan machine and 25 °C assayed manually (Figure 1). The mean lifespan was extended by 12.7% at 20 °C and by 8.5% at 25 °C. These data suggest that beyond correlation, Timut pepper extracts may play a more causal role in slowing aging.

### 3.2. Timut Pepper Extract Increases the Proportion of Worms That Are Active

**Figure 2:**
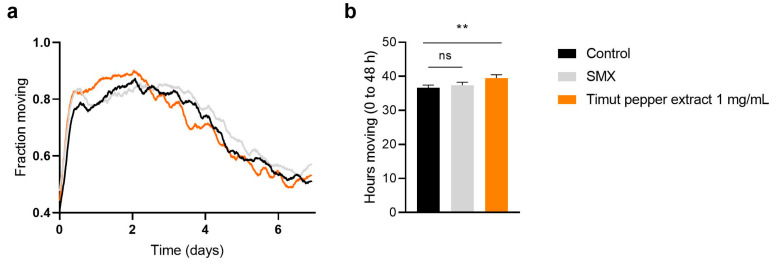
Fraction moving of control (water), SMX (sulfamethoxazole, positive control), and Timut pepper extract–treated worms. (**a**) Quantification of hours spent moving. (**b**) *C. elegans* strain SS104 glp-4(bn2). Statistical analysis: unpaired *t*-test. ns non-significant, ** *p* ≤ 0.01.

An increase in lifespan is desirable but only when it also prolongs health and mobility during aging. Similarly, to humans, during aging, *C. elegans* ceases to actively move and roam around [31]. To test this, we used the Wormgazer^TM^ imaging technology [30] and compared the effect of Timut pepper extract to water control and the positive control sulfonamide-antibiotic sulfamethoxazole (SMX), a compound known to extend health- and lifespan in *C. elegans* [28]. We found that *C. elegans* exposed to water (control) reached a plateau in fraction moving around day 1, and then a steady decline until day 7 (Figure 2a and Supplementary Video S1). Thus, in order to understand if Timut pepper extract could improve healthspan in addition to lifespan, we quantified the number of hours of moving by integrating the area under the curve for the moving fraction. Although *C. elegans* treated with the positive control SMX did not show any significant improvement within the time window from day 0 to day 2, compared to untreated control, *C. elegans* treated with 1 mg/mL Timut pepper extract had a higher fraction moving up to day 1.5. A significant increase in hours moving compared to control was observed in this time window (Figure 2b). Hence, in our findings, we observed a significant enhancement in the proportion of active C. elegans in young worms upon exposure to Timut pepper extract.

### 3.3. Timut Pepper Extract Enhances the Speed and Distance Moved of Worms, Including as They Age

**Figure 3:**
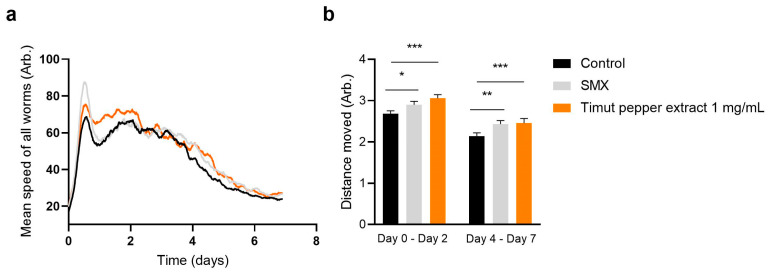
Mean speed of all *C. elegans* treated with control (water), SMX (sulfamethoxazole, positive control), and Timut pepper extract. (**a**) Quantification of distance moved. (**b**) *C. elegans* strain SS104 *glp-4(bn2)*. Statistical analysis: unpaired *t*-test. * *p* ≤ 0.05, ** *p* ≤ 0.01, *** *p* ≤ 0.001.

Next, we asked whether Timut pepper extract would improve not only the proportion of active worms, but also the intensity of such activity. In fact, speed has been shown to decline with age and correlate well with longevity [30,33]. Hence, we quantified the speed and distance moved by *C. elegans* exposed to water (control) or Timut pepper extract. When treated with 1 mg/mL Timut pepper extract, the speed of all *C. elegans* was higher than water control up to day 2, and again between day 4 and day 5 (Figure 3a and Supplementary Video S2). Further, integrating the area under the curve of the graph of the mean speed of all worms showed there was a significant increase in distance moved compared to control between day 0 and day 2 and between day 4 and day 7 (Figure 3b). Between day 2 and day 4, the speed and distance moved were similar to control (Figure 3a). Overall, the positive effects on speed and distance moved were comparable to the positive control SMX, showing that Timut pepper extract increases movement as the worms age, which is consistent with a greater healthspan.

### 3.4. Timut Pepper Extract Induces col-144 Expression

**Figure 4:**
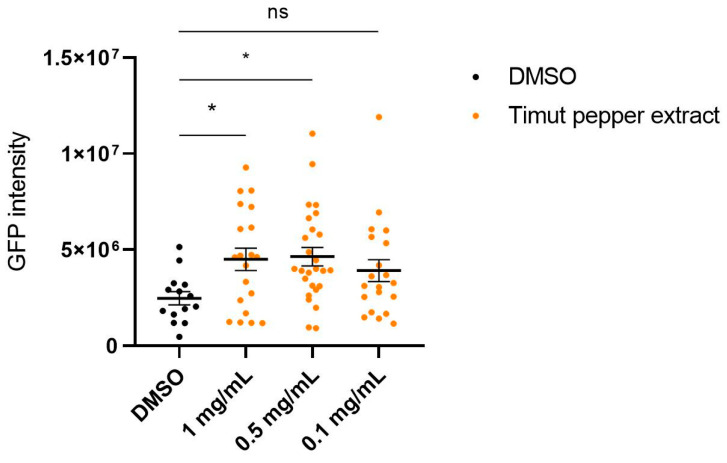
*col-144*::GFP intensity score for *C. elegans* strain LSD2002 grown on DMSO (vehicle control) or on different concentrations of Timut pepper extract. Statistical analysis: one-way ANOVA. * *p* ≤ 0.05, ns non-significant.

Besides decline in activity during aging, tissue morphology and integrity also decline. For instance, during aging, collagen mass declines in humans and in *C. elegans* [34,35]. To assess whether Timut pepper could slow down the age-dependent progressive loss of collagen expression, we used the reporter strain LSD2002 (*Pcol-144*::GFP) [32]. Exposure to Timut pepper extract resulted in higher collagen expression compared to DMSO control on day 4 of adulthood. The concentrations of 1 mg/mL and 0.5 mg/mL are statistically significantly different from the DMSO control (*p* = 0.0392 and *p* = 0.0181, respectively, one-way ANOVA). At 0.1 mg/mL, there was no significant difference detected (Figure 4). This suggests that Timut pepper extract prolongs the expression of collagens and slows the age-dependent collagen decline.

## 4. Discussion

Quality of life as we get older is an important concern for our aging society. The prevention of aging-related pathologies can be addressed in different ways, one of which is dietary interventions, in the form of healthier dietary habits or also through supplementation of beneficial micronutrients. Here, we investigated the lifespan- and healthspan-promoting potential of a Nepalese pepper extract of traditional use in the Himalayan region. We showed that exposure to Timut pepper extract consistently extends *C. elegans’* lifespan at 20 °C, as well as at 25 °C.

**Figure 5:**
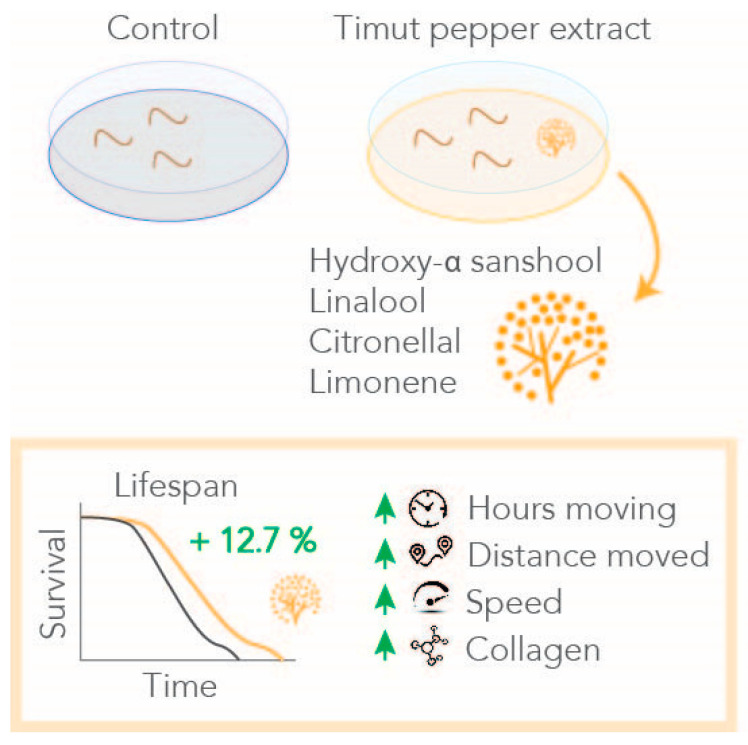
Timut pepper extract improves motility, boosts collagen expression, and extends lifespan of *C. elegans*.

Just days into adulthood, *C. elegans* experience a decline in optimal health, indicating that the aging process commences well before death. In their final week, *C. elegans* barely move and stop eating [33]. Exposure to Timut pepper extract could extend the proportion of *C. elegans* in active movement during the first days of adulthood, hinting towards an energy-enhancing effect of the extract. Maximum velocity has been shown to correlate with the longevity and healthspan of *C. elegans* previously [36], as a progressive decay in motor activity throughout lifespan has been observed [30,37]. In line with these studies and with our findings on active movement, distance moved and speed were also enhanced in the first week of adulthood of nematodes treated with Timut pepper extract, pointing to extended healthspan (Figure 5).

It would be of interest to expand the number of readouts on other locomotory behaviors affected by treatment with Timut pepper extract. For example, thrashing assays can be used as an alternative method to monitor nematodes’ motility [38], while chemotaxis assays are used to investigate nematodes’ learning and memory ability [39].

Furthermore, additional studies are needed to clarify the exact mechanism by which Timut pepper extract leads to an increase in locomotory performance of *C. elegans*. Transcriptome or proteome analysis could be used to gain an overview of the biochemical signaling pathways affected by treatment with Timut pepper extract. Indeed, several compounds which are contained in the extract could be responsible for its effect. The phytochemical composition of various Timut pepper extracts has been characterized, and alkaloids, lignins, sterols, and steroids, as well as amides, coumarins, carbonyl, and aromatic compounds, have been described as constituents of different plant parts [10]. The use of MCT oil in the extraction process of Timut pepper ensures a high bioavailability of the active molecules contained in it, as MCTs passively diffuse from the gastrointestinal tract into the blood. MCT oil is metabolized to ketone bodies that serve as an alternative source of energy for neurons, which may eventually improve cognitive and memory function [40]. Furthermore, fragrant compounds contained in Timut pepper, such as linalool, citronellal, and limonene, are known to have anxiolytic [41], antidepressant [42], anticonvulsant [43], and antinociceptive effects [44]. Linalool showed potential in improving cognitive performance of Alzheimer’s disease models [45,46], with a mechanism potentially involving the decrease of acetylcholinesterase activity and an increased expression of brain-derived neurotrophic factor (BDNF) and the tropomyosin kinase B (TrkB) receptor [47]. Citronellal and its derivatives have been evaluated for their neuroprotective and anti-inflammatory activities and for their action on the glutamatergic system [48,49]. Hydroxy-α-sanshool has been described as a major component of Timut pepper extracts [50] and has been shown to activate TRPV1 and TRPA1 channels in sensory neurons, thereby increasing the release of neurotransmitters [51] and eliciting their unique pungent, tingling sensation [8]. In addition, this compound has been described as inhibiting pH- and anesthetic-sensitive two-pore potassium channels (KCNK3, KCNK9, and KCNK18) [52].

A randomized, double-blind, placebo-controlled study on healthy volunteers was performed, evaluating cognitive performance on a hydroxy-α-sanshool standardized Timut pepper extract supplementation [53]. Here, treatments comprised four dark-brown soft gel capsules/day to be taken for 56 days. The capsules would contain either placebo or 2.8 g *Zanthoxylum armatum* DC. MCT oil extract (corresponding to 80 mg *Z. armatum* DC. extract). In this study, an increase in the speed of performing tasks and a concomitant reduction in hemodynamic responses in the frontal cortex during task performance of the supplemented subjects was observed. These findings suggest an effect of Timut pepper extract in increasing neural efficiency in humans [53]. The molecular mechanisms of cognition, aging, and longevity are very closely related, as neural stimulants/nootropics are reported to extend lifespan in various organisms and neurotransmitter signaling is discussed as one key mechanism to extend lifespan in *C. elegans* [54]. Typically, neurotransmitter signaling will slow down as a natural aging process and is enforced by unhealthy lifestyles [55,56,57].

## 5. Conclusions

In summary, we demonstrated that Timut pepper extract increases health- and lifespan of *C. elegans*, providing preliminary evidence of its potential benefits in the human diet.

## Data Availability

Data are contained within this article and Supplementary Materials.

## Supplementary Materials

The following supporting information can be downloaded at https://www.mdpi.com/article/10.3390/nu16132122/s1. Video S1: C elegans fraction moving and mean hours moving. Video S2: Mean speed and distance moved.
